# Regulation of immune cell responses by semaphorins and their receptors

**DOI:** 10.1186/ar3600

**Published:** 2012-02-09

**Authors:** Atsushi Kumanogoh, Hyota Takamatsu

**Affiliations:** 1 Department of Respiratory Medicine, Allergy and Rheumatic Diseases, Graduate School of Medicine Osaka University, 2-2 Yamada-oka, Suita, Osaka 565-0871, Japan; 2 World Premier International Immunology Frontier Research Center, Osaka University, 3-1 Yamada-oka, Suita, Osaka 565-0871, Japan

## Background

**Figure 1:**
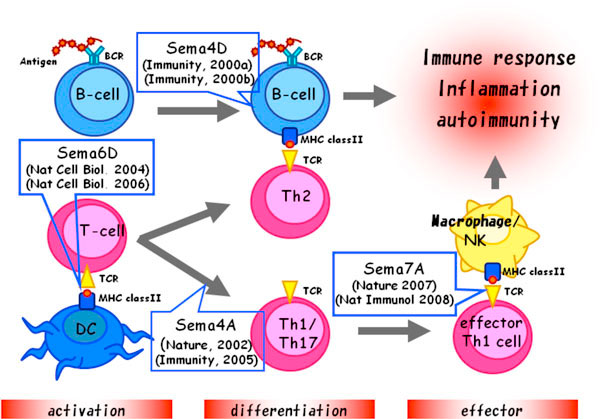
**Semaphorins are involved in physiological and pathological immune responses**.

Semaphorins were originally identified as axon guidance factors involved in the development of the neuronal system. However, accumulating evidence indicates that several members of semaphorins, so-called 'immune semaphorins', are crucially involved in various phases of immune responses (Figure 1) [1-3]. In addition, semaphorins and their receptors have been shown to be crucial for the pathogenesis of immunological disorders such as atopic dermatitis, multiple sclerosis, systemic sclerosis, systemic lupus erythematosus and rheumatoid arthritis, These semaphorins regulate immune cell interactions during physiological and pathological immune responses. However, conventional static analysis could not determine definitively whether they regulate immune cell movement.

## Materials and methods

Plexin-A1-/- mice were previously established [4]. Combinational studies, including imaging technique for visualizing single-cell dynamics and conventional immunological assays were performed.

## Results and discussion

**Figure 2:**
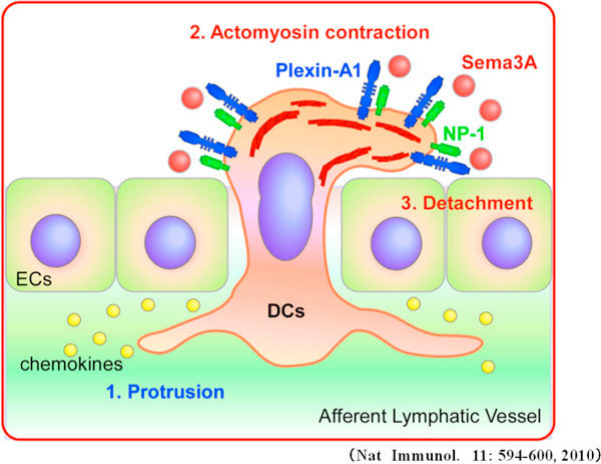
**Sema3A produced by the lymphatic induces actomyosin contraction during transmigration**.

We find that plexin-A1-mediated semaphorin signals are crucially involved in the transmigration of DCs across the lymphatics to exit the periphery to induce antigen-specific T-cell priming using plexin-A1-/- mice. In addition, adoptive transfer experiments identify that Sema3A produced in the lymphatics functions as a ligand for the plexin-A1/NP-1 receptor complex expressed in DCs. Interestingly, plexin-A1 is localized at the trailing edge but not the leading edge of DCs during migration. Sema3A induces phosphorylation of the myosin light chain to promote actomyosin contraction, resulting in increased DC velocity in the constricted area (Figure 2). Collectively, these findings not only demonstrate the involvement of semaphorins in immune cell trafficking but also indicate that semaphorins are therapeutic targets to treat immunological disorders.
